# Increased Red Blood Cell Distribution Width Associates with Cancer Stage and Prognosis in Patients with Lung Cancer

**DOI:** 10.1371/journal.pone.0080240

**Published:** 2013-11-11

**Authors:** Yasuko Koma, Akira Onishi, Hirofumi Matsuoka, Nao Oda, Naoya Yokota, Yusuke Matsumoto, Midori Koyama, Nobuhiko Okada, Nariyasu Nakashima, Daiki Masuya, Harukazu Yoshimatsu, Yujiro Suzuki

**Affiliations:** 1 Respiratory Center, Shinko Hospital, Kobe-city, Hyogo, Japan; 2 Department of Health Promotion & Human Behavior, Kyoto University Graduate School of Medicine / School of Public Health, Kyoto-city, Kyoto, Japan; National Cancer Center, Japan

## Abstract

**Background:**

Red cell distribution width (RDW), one of many routinely examined parameters, shows the heterogeneity in erythrocyte size. We investigated the association of RDW levels with clinical parameters and prognosis of lung cancer patients.

**Methods:**

Clinical and laboratory data from 332 patients with lung cancer in a single institution were retrospectively studied by univariate analysis. Kaplan-Meier survival analysis and Cox proportional hazard models were used to examine the effect of RDW on survival.

**Results:**

The RDW levels were divided into two groups: high RDW (>=15%), n=73 vs. low RDW, n=259 (<15%). Univariate analysis showed that there were significant associations of high RDW values with cancer stage, performance status, presence of other disease, white blood cell count, hemoglobin, mean corpuscular volume, platelet count, albumin level, C-reactive protein level, and cytokeratin 19 fragment level. Kruskal-Wallis tests revealed an association of RDW values with cancer stage in patients irrespective of comorbidity (patient with/without comorbidity: *p*<0.0001, patient without comorbidity: *p*<0.0001). Stages I-IV lung cancer patients with higher RDW values had poorer prognoses than those with lower RDW values (Wilcoxon test: *p*=0.002). In particular, the survival rates of stage I and II patients (n=141) were lower in the high RDW group (n=19) than in the low RDW group (n=122) (Wilcoxon test: *p*<0.001). Moreover, multivariate analysis showed higher RDW is a significant prognostic factor (*p*=0.040).

**Conclusion:**

RDW is associated with several factors that reflect inflammation and malnutrition in lung cancer patients. Moreover, high levels of RDW are associated with poor survival. RDW might be used as a new and convenient marker to determine a patient’s general condition and to predict the mortality risk of lung cancer patients.

## Introduction

Red cell distribution width (RDW) is routinely examined with the complete blood count (CBC) test that shows the heterogeneity in erythrocyte size. It is used clinically to differentiate different types of anemia. Recent studies have reported the association between high RDW levels and increased mortality in the general population [1,2], in patients with cardiovascular disease [3,4], brain vascular disease [5], septicemia [6], chronic obstructive pulmonary disease [7], and hepatitis B [8]. Mortality, the activity of inflammatory bowel disease [9], and pulmonary functions [10] have also been reported to be associated with RDW values. The mechanism underlying those associations of RDW with survival or disease activity has not been elucidated, but high levels of RDW are thought to be provoked by chronic inflammation, poor nutritional status (e.g. iron, folate, and vitamin B12 deficiency), and age-associated diseases via changes in erythropoiesis [1,11,12].

Malignant tumors are known to evoke chronic inflammation and malnutrition [13]. The resulting low performance status (PS) and compromised nutritional status account for the patient’s poor quality of life, and are obstacles in cancer treatment. Generally, markers such as age, PS and disease stage are used to risk-stratify patients and guide therapeutic strategy. These parameters provide some useful information, but the clinical conditions of cancer patients are so complex that finding biomarkers to define the patient’s general condition remains a significant challenge.

We hypothesized that RDW levels might be associated with cancer progression because RDW reflects chronic inflammation and nutritional status. There are few reports on the relationship between RDW and malignant tumors [14-19], and they are limited to small studies that distinguish malignant from benign tumors, or predict a malignant tumor. In particular, there has been no specific study on the relationship of RDW with cancer stages or patient survival. Therefore, in this study, we investigated RDW levels in lung cancer patients and determined the relationships between RDW and various clinical parameters, including survival in lung cancer patients. 

## Methods

### Study subjects

Patients diagnosed with lung cancer in Shinko hospital between May 2008 and September 2012 were retrospectively studied. This study was approved by the institutional review board (IRB) of Shinko Hospital and written consent was waived by the approving IRB. The diagnosis of lung cancer was made pathologically with bronchoscopic biopsy, CT-guided needle lung biopsy, or surgically resected specimen. The cancer stage was determined in accordance with the TNM (tumor-node-metastasis) classification system (International Union against Cancer; UICC-7). PS was quantified using the World Health Organization approved Eastern Cooperative Oncology Group (ECOG) PS scale. Blood samples were collected from patients using routine methods at the time of diagnosis. Blood parameters, white blood cell (WBC), hemoglobin, mean cell volume (MCV), and RDW were analyzed with an automated hematology analyzer XE-5000 (Sysmex Corporation, Kobe, Japan). The normal range for RDW in general and in our laboratory is 11.5% to 15% [8,20]. Biochemical factors, albumin, creatinine, and C-reactive protein (CRP) were analyzed with a TBA™-c16000 (Toshiba Medical Systems Corporation, Ohtawara, Japan). Tumor markers were analyzed with an Architect Analyzer i2000SR (Abbot, IL, USA). 

### Statistical analysis

Continuous variables were expressed as mean value ±standard deviation (SD). We used JMP version 6 (SAS Institute Inc., Cary, NC, USA) to perform statistical procedures. Differences in categorical factors were determined with Fisher’s exact test. Normality was assessed by Shapiro-Wilk goodness-of-fit tests. Differences in continuous values between two groups were assessed with Student’s *t* test for normally distributed variables, and non-parametric Mann-Whitney *U* tests for non-normally distributed variables as appropriate. Differences in continuous variables among three or more groups were assessed with one-way analysis of variance for normally distributed variables and Kruskal-Wallis for non-normally distributed variables. The Tukey-Kramer test was used for adjustment for multiple testing [21]. Survival rate curves were drawn according to the Kaplan-Meier method, and differences between curves were analyzed by the Wilcoxon test. Univariate and multivariate Cox proportional hazard model were performed for significance of prognostic variables. The date of diagnosis was considered day zero. The terminal event for survival was cancer-related death of the patients. A *p*-value <0.05 was considered statistically significant.

## Results

### Study subjects

The study included 332 lung cancer patients in total; their characteristics are shown in Table 1. The range in the RDW in the study was 11.9 to 23.5 with a median of 14.3. At the time of diagnosis, 195 patients had comorbid diseases, including lung disease (chronic obstructive pulmonary disease, tuberculosis sequelae), liver disease (hepatitis B and C, alcoholic liver injury), heart disease (heart failure, arrhythmia), diabetes and others, and the remaining 137 patients did not have any other diseases. 

**Table 1:** Patient characteristics.

	**Patients**
No.	332
Mean age (years, range)	71.5 (38-94)
Gender (male/female)	223/109
Median BI (range)	889 (0-4400)
ECOG PS (0/1/2/3/4)	94/161/54/20/3
Stage (I / II / III / IV)	102/39/79/112
Other diseases* (y/n)	195/137
Lung disease	95
Liver disease	36
Heart disease	13
Diabetes	47
Other	36
Histology	
Adenocarcinoma	168
Squamous cell carcinoma	91
Adenosquamous carcinoma	8
Large cell carcinoma	13
Small cell carcinoma	32
Others	20

* Total number of patients with other diseases. Some patients had more than one disease.

BI, Brinkman index; ECOG PS, Eastern Cooperative Oncology Group Performance Status; y, yes; n, no.

### RDW values and clinical parameters

Patients were divided into two groups based on their RDW values. The upper normal range (15%) was used as a boundary: 73 patients had a high RDW (≧15%) and 259 patients had a low RDW (<15%). Clinical and laboratory characteristics of these groups are summarized in Table 2. The patients with higher RDW values had more advanced cancer stages (*p*=0.005), poorer PS (*p*=0.001), other diseases (*p*=0.006), higher WBC counts (*p*<0.001), lower hemoglobin levels (*p*<0.0001), lower MCV (*p*<0.001), higher platelet counts (*p*=0.045), lower albumin levels (*p*<0.0001), higher CRP levels (*p*<0.001), and higher cytokeratin 19 fragment (CYFRA) levels (*p*<0.001). 

**Table 2:** Clinical and laboratory data from lung cancer patients.

**Variables**	**RDW<15(%)**		**RDW≧15(%)**		**n**	***p*-value**
		**n**		**n**		
Age (years,±SD)	71.1±9.7	259	72.8±10.6	73	332	0.16
Gender (male/female)	172/87	259	51/22	73	332	0.67
Stage (I/II/III/IV)	89/33/61/76	259	13/6/18/36	73	332	0.005
Smoking (BI)	840 (0-1380)	259	980 (450-1245)	73	332	0.55
ECOG PS (0/1/2/3/4)	84/125/37/12/1	259	10/36/17/8/2	73	332	0.001
Other diseases (y/n)	142/117	259	53/20	73	332	0.006
Biochemical parameters						
WBC (/μL)	7000 (5900-8600)	259	8200 (6650-10750)	73	332	<0.001
Hemoglobin (g/dL)	13.4 (12.5-14.4)	259	12.0 (10.4-13.3)	73	332	<0.0001
MCV (fL)	93.6 (90.1-96.3)	259	90.2 (84.3-94.6)	73	332	<0.001
Platelet (10^4^/μL)	23.6 (20.0-30.2)	259	26.4 (20.8-34.3)	73	332	0.045
Albumin (g/dL)	4.2 (3.9-4.4)	259	3.8 (3.5-4.1)	73	332	<0.0001
Creatinine (mmol/L)	0.77 (0.65-0.89)	259	0.77 (0.61-0.94)	73	332	0.95
CRP (mg/dL)	0.26 (0.07-1.75)	259	1.22 (0.23-3.64)	73	332	<0.001
Tumor markers						
CEA (ng/mL)	4.5 (3.0-12.3)	255	6.1 (3.4-12.2)	70	325	0.15
CYFRA (ng/mL)	2.8 (1.7-5.4)	239	4.7 (2.7-10.3)	69	308	<0.001
NSE (ng/mL)	11.4 (9.2-16.0)	200	11.4 (8.9-19.2)	54	254	0.71
ProGRP (pg/mL)	27.3 (20.8-41.2)	226	28.6 (22.3-42.7)	67	293	0.68

Data are presented as mean ± SD or median (first quartile-third quartile). RDW, red cell distribution width; SD, standard deviation; BI, Brinkman index; ECOG PS, Eastern Cooperative Oncology Group Performance Status; y, yes; n, no, WBC, white blood cell; MCV, mean cell volume; CRP, C-reactive protein; CEA, carcinoembryonic antigen; CYFRA, cytokeratin 19 fragment; NSE, neuron specific enolase; ProGRP, pro-gastrin-releasing peptide.

A number of diseases are reported to be associated with RDW values [3-10]. As such, we next excluded the 195 patients with any comorbidities, and the remaining 137 patients were divided into two groups: 20 patients with high RDW (≧15%) and 117 patients with low RDW (<15%) (Table 3). The patients with higher RDW values had more advanced cancer stages (*p*<0.0001), poorer PS (*p*<0.0001), higher WBC counts (*p*=0.006), lower hemoglobin levels (*p*<0.0001), lower MCV (*p*<0.0001), higher platelet counts (*p*=0.019), lower albumin levels (*p*<0.0001), higher CRP levels (*p*=0.002), and higher CYFRA levels (*p*=0.002). In addition, age was associated with RDW values (*p*=0.046). 

**Table 3:** Clinical and laboratory data from lung cancer patients without any other disease.

**Variables**	**RDW<15(%)**		**RDW≧15(%)**		**n**	***p*-value**
		**n**		**n**		
Age (years,±SD)	69.4±10.6	117	74.1±13.1	20	137	0.046
Gender (male/female)	70/47	117	9/11	20	137	0.23
Stage (I/II/III/IV)	42/13/24/38	117	0/0/4/16	20	137	<0.0001
Smoking (BI)	540 (0-1035)	117	400 (0-1075)	20	137	0.52
EOCG PS (0/1/2/3/4)	43/55/14/5/0	117	3/5/5/5/2	20	137	<0.0001
Biochemical parameters						
WBC (/μL)	7000 (5950-8200)	117	8550 (6825-13975)	20	137	0.006
Hemoglobin (g/dL,)	13.4 (12.7-14.5)	117	11.1 (10.1-13.5)	20	137	<0.0001
MCV (fL)	92.7 (89.7-95.9)	117	88.5 (82.8-92.7)	20	137	<0.0001
Platelet (10^4^/μL)	24.4 (20.7-31.4)	117	28.7 (24.3-37.6)	20	137	0.019
Albumin (g/dL)	4.2 (4.0-4.4)	117	3.8 (3.5-4.1)	20	137	<0.0001
Creatinine (mmol/L)	0.74 (0.64-0.88)	117	0.81 (0.63-1.05)	20	137	0.49
CRP (mg/dL)	0.18 (0.06-1.13)	117	1.58 (0.28-5.46)	20	137	0.002
Tumor markers						
CEA (ng/mL)	4.3 (3.0-12.7)	116	7.8 (2.7-19.0)	19	135	0.19
CYFRA (ng/mL)	2.4 (1.4-4.6)	111	5.0 (3.2-17.2)	19	130	0.002
NSE (ng/mL)	11.2 (8.8-15.7)	94	14.2 (8.9-27.3)	15	109	0.24
ProGRP (pg/mL)	27.7 (20.3-39.6)	108	29.7 (21.7-40.8)	18	126	0.86

Data are presented as mean ± SD or median (first quartile-third quartile). RDW, red cell distribution width; SD, standard deviation; BI, Brinkman index; ECOG PS, Eastern Cooperative Oncology Group Performance Status; WBC, white blood cell; MCV, mean cell volume; CRP, C-reactive protein; CEA, carcinoembryonic antigen; CYFRA, cytokeratin 19 fragment; NSE, neuron specific enolase; ProGRP, pro-gastrin-releasing peptide.

### RDW values and cancer stages

**Figure 1:**
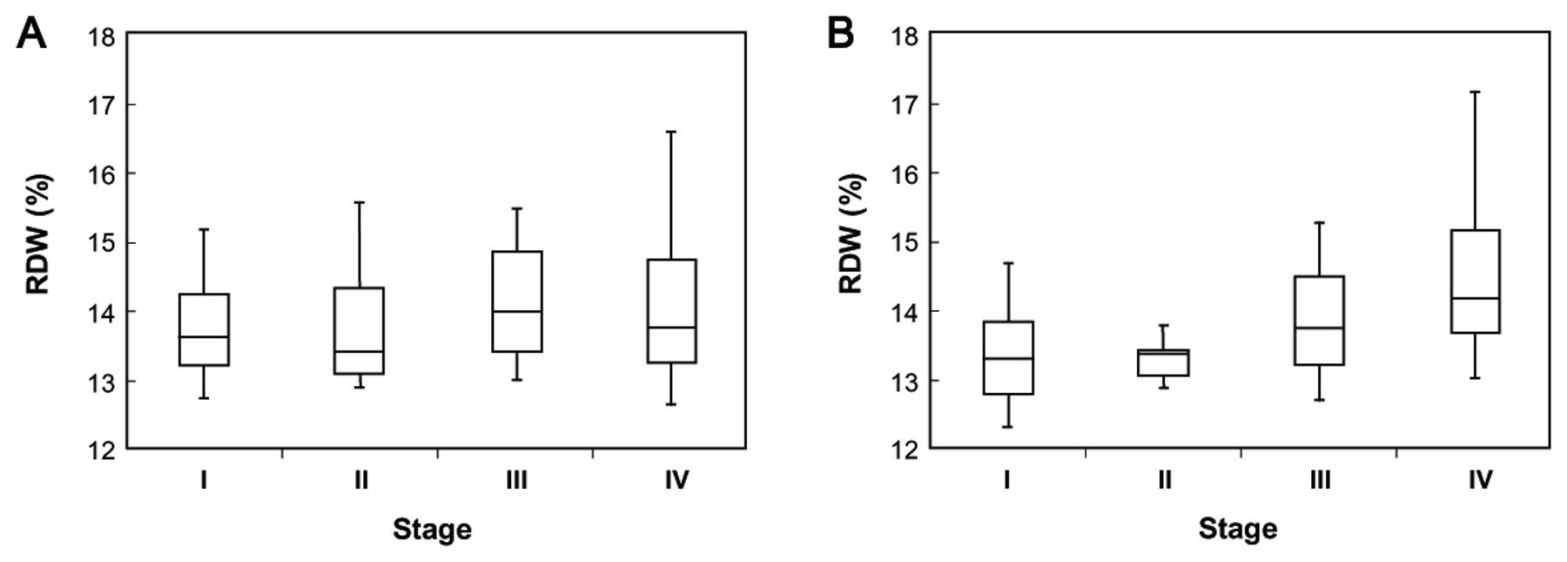
RDW levels in lung cancer patients according to stage. (A) RDW levels of lung cancer patients. Kruskal-Wallis test: *p*<0.0001. Tukey non-parametric test: *p*-value I–II=0.58; I–III=0.039; I–IV<0.001; II–III=0.29; II–IV=0.021; III–IV=0.13. (B) RDW levels of patients with lung cancer without any comorbid disease. Kruskal-Wallis test: *p*<0.0001. Ad hoc, Tukey non-parametric test: *p*-value I–II=0.96; I–III=0.043; I–IV=<0.0001; II–III=0.13; II–IV<0.0001; III–IV<0.001. The box plots in the figure represent columns of data as boxes whose extents indicate the 25^th^ and 75^th^ percentiles. The line inside the box represents the median. Capped bars indicate the minimum and maximum values.

Patient RDW values according to cancer stages were examined, and we found an association between cancer stage and RDW (*p*<0.0001, Kruskal-Wallis test, Figure 1A). The RDW values were also associated with cancer stage in patients without any other diseases (*p*<0.0001, Kruskal-Wallis test, Figure 1B). 

### RDW values and prognosis

**Figure 2:**
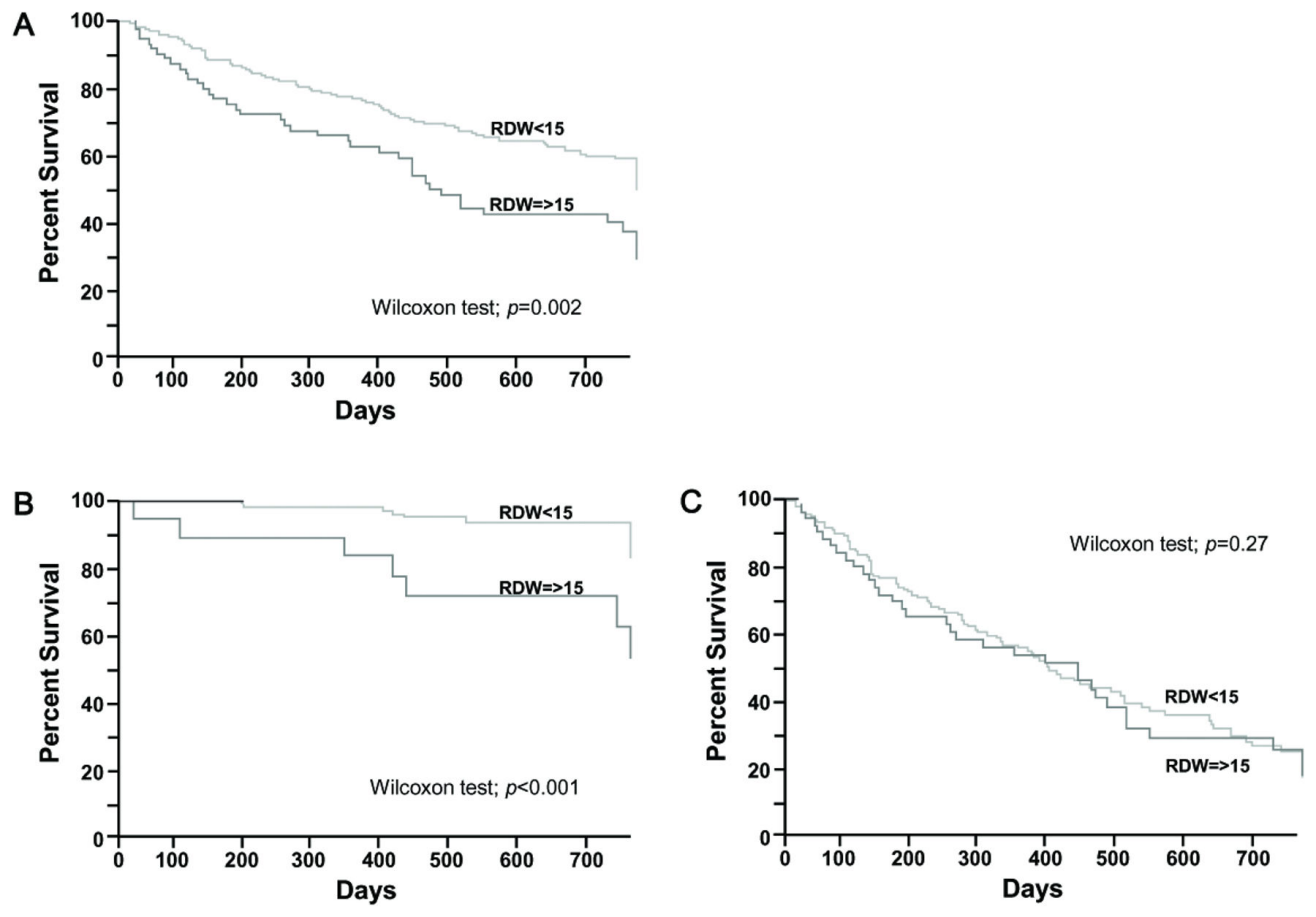
Survival rates of lung cancer patients stratified by RDW. (A) Survival rates for patients with stages I-IV lung cancer (n=332, high RDW (n=73) vs low RDW (n=259)). (B) Survival rate for patients with stages I and II lung cancer (n=141, high RDW (n=19) vs. low RDW (n=122)). (C) Survival rate for patients with stages III and IV lung cancer (n=191, high RDW (n=54) vs. low RDW (n=137)).

We performed a prognostic study of 332 patients followed up for 24 months. Within the group, 125 patients died from lung cancer-related causes. The survival rate is shown in Figure 2. As shown in Figure 2A, groups with all stages of lung cancer having high RDW values had a worse prognosis than those with low RDW values (Wilcoxon test: *p*=0.002). 

Next, we divided 332 patients into two groups: early stages of lung cancer (stages I and II, n=141, Figure 2B) and progressive stages of lung cancer (stages III and IV, n=191, Figure 2C). As shown Figure 2B, among the patients with early stages of lung cancer, the patients with high RDW values had a worse prognosis (Wilcoxon test: *p*<0.001). Meanwhile, no statistically significant differences were observed between the two groups in patients with progressed lung cancer stages (Figure 2C, Wilcoxon test; *p*=0.27).

The results of the Cox regression analysis are presented in Table 4. The group with higher RDW values showed significant differences (hazard ratio=2.15, 95% confidence interval 1.04-4.46, *p*=0.040) when we controlled for stage, PS, presence of other disease, presence of treatment, albumin, CRP, and interaction of stage and RDW. We also identified borderline significant interaction between RDW values and cancer stage (*p*=0.062), which was consistent with subgroup analysis based on cancer stage (early stages and progressive stages).

**Table 4:** Univariate and Multivariate Cox proportional hazard model for survival (n=332).

**Variables**	**Univariate analysis**		**Multivariate analysis**	
	**HR (95% CI)**	***p*-value**	**HR (95% CI)**	***p*-value**
RDW ≧15 vs <15 (%)	1.33 (1.10-1.59)	0.003	2.15 (1.04-4.46)	0.040
Stage		<0.0001		<0.0001
II vs I	2.80 (1.13-7.05)		2.18 (0.70-6.12)	
III vs II	3.63 (1.90-7.67)		2.82 (1.29-7.75)	
IV vs III	1.57 (1.09-2.29)		1.39 (0.88-2.22)	
ECOG PS		<0.0001		<0.001
1 vs 0	1.81 (1.14-3.01)		1.88 (1.16-3.14)	
2 vs 1	2.63 (1.72-3.96)		1.82 (1.16-2.81)	
3 vs 2	1.68 (0.89-3.04)		0.67 (0.34-1.25)	
4 vs 3	4.05 (0.93-12.6)		3.91 (0.84-13.4)	
Other diseases y vs n	1.19 (0.85-1.66)	0.31	0.94 (0.78-1.14)	0.55
Treatment y vs n	0.43 (0.35-0.53)	<0.0001	0.53 (0.43-0.67)	<0.0001
Albumin	0.27 (0.19-0.38)	<0.0001	0.54 (0.33-0.90)	0.019
CRP	1.14 (1.11-1.18)	<0.0001	1.05 (1.00-1.10)	0.033
Stage*RDW		0.001		0.062

HR, hazards ratio; CI, confidence interval; RDW, red cell distribution width; ECOG PS, Eastern Cooperative Oncology Group Performance Status; CRP, C-reactive protein; y, yes; n, no.

## Discussion

To our knowledge, the present study is the first to analyze RDW in lung cancer patients. We revealed two major findings: (1) RDW values were positively associated with clinical cancer stage; and (2) patients with higher RDW values had poorer prognoses. 

As shown in Tables 2 and 3, advanced stage, poorer PS, high WBC, lower albumin, and higher CRP were associated with high RDW. This result might support the idea that high levels of RDW reflect chronic inflammation and poor nutritional status of patients with lung cancer. In addition, the presence of comorbid diseases, which might provoke inflammation and poor nutritional status, was also associated with RDW values (Table 2), implying that comorbidity might also contribute to RDW values. This result is in accordance with previous reports that showed an association of high RDW values and some diseases [3-10]. Next, as shown in Figure 1, the results of Kruskal-Wallis tests revealed a positive association between clinical cancer stage and the level of RDW (Figure 1A, B). This result might also reflect an association between RDW and increased inflammation or malnutrition induced by cancer progression.

Next, we revealed a significant association between high RDW and poor prognosis of cases within any stage of lung cancer (Figure 2A). Because RDW values are positively associated with cancer stage as shown in Figure 1, it is not surprising that univariate analysis performed on patients in any stage showed that patients with higher RDW values had shorter survival. Therefore, we divided cancer stages into two groups: the early stage group (stage I, II) and progressed stage group (stage III, IV), to exclude the stage effect on survival rate (Figure 2B, C). When cancer stage was aligned, the group with higher RDW values had a significantly poorer prognosis in the early-stage group. Naturally, there are differences in prognosis in each case even if they are in the same cancer stage, because many confounding factors are involved in survival. One explanation for the difference in prognosis in early-staged patients might be the strong association of RDW value with PS, albumin, and CRP. Poorer survival in patients with poor PS, low levels of albumin, and higher levels of CRP is well established [22-26], and our study indicated similar results (Table 4), showing that they are independent prognostic factors. In addition to these factors, RDW was also an independent prognostic factor. One merit of RDW assessment is that it can be described without any human bias, whereas PS scores rely on a clinician’s perception of a patient’s condition.

Inflammation also impacts patient survival [25]. The exact mechanisms of inflammation on RDW levels are unknown, but potential mechanisms include impairing iron metabolism, inhibiting the response to erythropoietin, and decreasing red blood cell survival via production of inflammatory markers [27,28]. The overproduction of selective cytokines such as interleukin-6, tumor necrosis factor α, and CRP has been shown to play a key role in inducing chronic inflammation in cancer patients [27]. In addition, chronic inflammation is also reported to lead to a poor response to chemotherapy [22]. Hence the poorer survival in patients with higher RDW values might be due to chronic inflammation itself, or the poor response to chemotherapy; however, further investigation is needed to explain the relationships of RDW with inflammation and the response to cancer treatment. 

The study subjects in the prognostic analysis included those with all types of histology, and the presence or absence of cancer therapy was not distinguished. Generally, patients with the initial stage of non-small cell lung carcinoma (NSCLC) are best treated with surgery when possible. However, palliative chemotherapy, radiotherapy or even best supportive care is sometimes chosen depending on the patient’s general condition. Decision-making in cancer treatment strongly relies on patient status and complex factors, such as nutritional status, PS, and the presence of comorbid disease. Since the cases with high RDW values reflected each patient’s poorer general condition, best supportive care instead of radical operation or chemotherapy might tend to be chosen for them. Meanwhile, there was no significant difference in prognosis in patients with stage III and IV lung cancer, indicating that many other factors not directly reflected by RDW values play a greater role in survival when cancer has progressed.

There are some limitations in this study. For instance, the relationships between RDW and histological types were not made clear in this study. Lung cancer is classified into two major histological types, small cell lung carcinomas and NSCLC, and the clinical course and response to treatment greatly vary depending on the histology. There was no differential association of RDW levels across lung cancer histology (not shown). Since the number of the each histological type was small in this study, further investigation is needed to determine the associations of RDW and lung cancer histology.

Because we considered a large number of factors in analyses, multiple comparisons are necessary in a confirmatory study. However, our study was an exploratory one to search for any factors associated with RDW values. If adjustments for multiple comparisons were made in exploratory studies, important associated factors would be missed. Because adjustments for multiple comparisons were not made in our study (as in a number of previous exploratory studies) it will be necessary to validate factors identified in our study in future confirmatory studies.

In conclusion, this study revealed an association of RDW values with PS, albumin levels, WBC counts, platelet counts, the presence of comorbid disease and clinical cancer stage. Moreover, significant associations between higher RDW levels and poor survival, especially in patients with early stage lung cancer, were also shown. In cancer treatment, as patients’ clinical courses vary and are difficult to predict, stratification of patients for appropriate surveillance programs and different tailored therapeutic strategies is important. Because RDW values can be routinely examined by CBC tests, RDW might be a new and convenient marker to understand the patient’s general condition and to predict the mortality risk of a patient within a determined lung cancer stage. 
